# Identification and Characterization of Neuropeptides by Transcriptome and Proteome Analyses in a Bivalve Mollusc *Patinopecten yessoensis*

**DOI:** 10.3389/fgene.2018.00197

**Published:** 2018-06-05

**Authors:** Meiwei Zhang, Yangfan Wang, Yangping Li, Wanru Li, Ruojiao Li, Xinran Xie, Shi Wang, Xiaoli Hu, Lingling Zhang, Zhenmin Bao

**Affiliations:** ^1^MOE Key Laboratory of Marine Genetics and Breeding, Ocean University of China, Qingdao, China; ^2^Laboratory for Marine Biology and Biotechnology, Qingdao National Laboratory for Marine Science and Technology, Qingdao, China; ^3^Laboratory for Marine Fisheries Science and Food Production Processes, Qingdao National Laboratory for Marine Science and Technology, Qingdao, China

**Keywords:** bivalve mollusc, neuropeptide, ganglia transcriptome, mass spectrometry, reproduction, cardioactivity, shell growth, eye functioning

## Abstract

Neuropeptides play essential roles in regulation of reproduction and growth in marine molluscs. But their function in marine bivalves – a group of animals of commercial importance – is largely unexplored due to the lack of systematic identification of these molecules. In this study, we sequenced and analyzed the transcriptome of nerve ganglia of Yesso scallop *Patinopecten yessoensis*, from which 63 neuropeptide genes were identified based on BLAST and *de novo* prediction approaches, and 31 were confirmed by proteomic analysis using the liquid chromatography-tandem mass spectrometry (LC-MS/MS). Fifty genes encode known neuropeptide precursors, of which 20 commonly exist in bilaterians and 30 are protostome specific. Three neuropeptides that have not yet been reported in bivalves were identified, including calcitonin/DH31, lymnokinin and pleurin. Characterization of glycoprotein hormones, insulin-like peptides, allatostatins, RFamides, and some reproduction, cardioactivity or feeding related neuropeptides reveals scallop neuropeptides have conserved molluscan neuropeptide domains, but some (e.g., GPB5, APGWamide and ELH) are characterized with bivalve-specific features. Thirteen potentially novel neuropeptides were identified, including 10 that may also exist in other protostomes, and 3 (GNamide, LRYamide, and Vamide) that may be scallop specific. In addition, we found neuropeptides potentially related to scallop shell growth and eye functioning. This study represents the first comprehensive identification of neuropeptides in scallop, and would contribute to a complete understanding on the roles of various neuropeptides in endocrine regulation in bivalve molluscs.

## Introduction

Neuropeptides are intercellular signaling molecules secreted by neurons, acting as hormones, neurotransmitters, and modulators. As modulators of neuronal activity, neuropeptides contribute to the generation of different outputs from the same neuronal circuit in a context dependent manner (Jékely, 2013), or organizing complex motor functions (Kim et al., 2006). They play key roles in regulating various physiological processes, including growth, metabolism, reproduction, etc. For example, insulin-like peptides can promote the growth of *Drosophila* (Slaidina et al., 2009), and regulate metabolism in *Aplysia* (Floyd et al., 1999). Feeding circuit-activating peptide is involved in the induction and maintenance of food-induced arousal (Sweedler et al., 2002). GnRH and kisspeptin participate in reproduction regulation in many vertebrates (Ottinger et al., 2002; Tsutsui et al., 2010; Gopurappilly et al., 2013).

Identification of neuropeptides represents the first step to unraveling the function of these molecules. Neuropeptides of interest are usually identified through high-performance liquid chromatography (HPLC) isolation combined with mass spectrometry (MS) (Santama et al., 1995b; Henry et al., 1997, 2000; Willoughby et al., 1999) or by gene cloning (Satake et al., 1999a,b; Iwakoshi et al., 2002). Novel neuropeptides can be identified using MS supported by genetic information such as genome or transcriptome sequences (Marvin et al., 2001; Zatylny-Gaudin et al., 2010; Nagasawa et al., 2015b). Recent advances in high-throughput sequencing facilitate comprehensive identification of neuropeptides, which can be obtained solely through transcriptome/genome prediction or in combination with MS. For example, based on the transcriptomes of starfish *Asterias rubens* (Semmens et al., 2016; Semmens and Elphick, 2017) and annelid *Platynereis dumerilii* (Conzelmann et al., 2013), 40 and 98 candidate neuropeptide precursors were identified, respectively. From the genomes of annelids *Capitella teleta* and *Helobdella robusta*, nematode *Caenorhabditis elegans* and arthropod *Drosophila melanogaster*, 30–43 neuropeptide precursors were predicted (Li et al., 1999; Nässel, 2002; Veenstra, 2011), and by integrating the genomic, transcriptomic and proteomic data, 48 and 73 neuropeptide precursors were identified from the starfish *Acanthaster planci* (Smith et al., 2017) and arthropod *Daphnia pulex* (Dircksen et al., 2011), respectively.

Mollusca is the most speciose phylum of Lophotrochozoa that widely distributed in water and on land. It includes three major subgroups: cephalopods, gastropods, and bivalves. Most neuropeptide research has been on the gastropod *Aplysia*, a well-established model organism for cellular and systems neural science (Moroz et al., 2006). Recently, by analyzing the genome and/or transcriptome databases, neuropeptidomes of another four gastropods, *Lottia gigantea* (Veenstra, 2010), *Charonia tritonis* (Bose et al., 2017), *Deroceras reticulatum* (Ahn et al., 2017) and *Theba pisana* (Adamson et al., 2015) were reported, which provide valuable resources for a more comprehensive understanding on the neuroendocrine regulation mechanisms in gastropods. In contrast, transcriptome- or genome-wide identification of neuropeptides is relatively scarce in bivalves. Till now, only one study was conducted which reports 74 putative neuropeptide genes from the genome and transcriptome databases of two oysters, *Pinctada fucata* and *Crassostrea gigas* (Stewart et al., 2014).

The Yesso scallop *Patinopecten yessoensis* is an important maricultural bivalve in both China and Japan. Due to its commercial importance, research on *P. yessoensis* primarily focuses on reproduction (Matsutani and Nomura, 1987; Osada et al., 2004), immunity (Zhang et al., 2013; Li R. et al., 2015; Ning et al., 2015; Wang et al., 2015; Zou et al., 2015) and metabolism (Zhang et al., 2014; Li X. et al., 2015). Several studies reveal that molecular signals from nerve ganglia play vital roles in scallop gonadal development. For example, it is reported that neurotransmitters, such as GABA and glycine, may participate in scallop ovary development (Li et al., 2016), and GnRH can stimulate spermatogonial proliferation (Nakamura et al., 2007) and inhibit oocyte growth (Nagasawa et al., 2015a). But till now, no systematic identification of neuropeptides has been performed. In this study, we interrogate the transcriptome and proteome of *P. yessoensis* nerve ganglia to comprehensively identify and characterize neuropeptide genes. This study provides a valuable resource for future research on the functioning of neuropeptides in bivalve molluscs.

## Materials and Methods

### Sample Collection

Two-year-old Yesso scallops *P. yessoensis* were obtained from the Dalian Zhangzidao Fishery Group Corporation (Liaoning Province, China) in January 2014. After collection, the scallops were acclimated at 8°C in aerated seawater for 1 week. After acclimation, three individuals were randomly chosen and their nerve ganglia were dissected, immediately frozen in liquid nitrogen and stored at -80°C before use.

### RNA Isolation, Transcriptome Sequencing, and Assembly

Total RNA of the pooled ganglia samples was extracted using the conventional guanidinium isothiocyanate method. RNA concentration and purity were determined using a Nanovue Plus spectrophotometer (GE Healthcare, Princeton, NJ, United States), and RNA integrity was verified by agarose gel electrophoresis. RNA-seq library was constructed using the NEBNext mRNA Library Prep Master Mix Set for Illumina according to the manufacturer’s instructions, and then subjected to paired-end sequencing of 100 bp on the Illumina HiSeq 2000.

Raw reads were first filtered using a homemade Perl script to remove the reads that contain more than five ambiguous bases (N) or 10 low-quality bases (base quality score less than 20). Then, the resulting high-quality (HQ) reads were assembled using Trinity (Grabherr et al., 2011) with the default parameters. The data have been submitted to the NCBI Sequence Read Archive under accession number SRP127306.

### Identification and Functional Annotation of Neuropeptide Precursors

To search for transcripts encoding putative neuropeptide or peptide hormone precursor proteins in *P. yessoensis*, the homologous sequences previously identified in other molluscs (*Achatina fulica*, *Aplysia californica*, *Aplysia kurodai*, *Argopecten irradians*, *Biomphalaria glabrata*, *Crassostrea gigas*, *Crassostrea virginica*, *Charonia tritonis*, *Conus victoriae*, *Euprymna scolopes*, *Fusinus ferrugineu*, *Haliotis asinina*, *Helix lucorum*, *Helix pomatia*, *Idiosepius notoides*, *Lingula anatina*, *Lottia gigantea*, *Lymnaea stagnalis*, *Mercenaria mercenaria*, *Mytilus edulis*, *Mytilus galloprovincialis*, *Octopus bimaculoides*, *Octopus vulgaris*, *Pinctada fucata*, *Pinctada maxima*, *Physa acuta*, *Sinonovacula constricta*, *Thais clavigera*, and *Theba pisana*) were downloaded and used as queries in tBLASTn searches of the assembled scallop transcriptome database with an *E*-value cutoff of 1e-4. Open reading frames (ORFs) of these potential neuropeptide sequences were identified using EMBOSS GUI v1.12: getorf. The resultant protein sequences were further assessed as potential precursors of secreted bioactive peptides by investigating: (i) the presence of a putative N-terminal signal peptide sequence using SignalP 4.1^1^ and Signal-3L 2.0^2^, (ii) the presence of putative monobasic or dibasic cleavage sites N-terminal and C-terminal to the putative bioactive peptides, with reference to known consensus cleavage motifs (Seidah and Chrétien, 1999; Veenstra, 2000; Liu et al., 2006), (iii) the presence of a C-terminal glycine residue that is a potential substrate for amidation, and (iv) the presence of cysteine residues which are likely to form disulfide bridges.

*De novo* prediction of neuropeptide precursors was performed by analyzing the assembled transcriptome sequences using a neuropeptide-prediction tool NpSearch, which searches for sequences with characteristics of neuropeptide precursors (signal peptide, cleavage sites, C-terminal glycine and repeated peptides)^3^.

Functional annotation of the identified neuropeptides was conducted by searching against NCBI non-redundant protein sequences (nr) database using BLASTx algorithm with the *E*-value threshold of 1e-5.

### Sequence Alignment

The neuropeptide homologous sequences were collected from GenBank. Multiple alignments were conducted using ClustalW (Thompson et al., 1994), and the results were annotated with GeneDoc (Nicholas and Nicholas, 1997). The frequency of each amino acid in the alignment result was presented using the online tool WebLogo (Crooks et al., 2004).

### Selective Pressure Analysis

Selective pressure analysis of the neuropeptide genes was conducted among *P. yessoensis* and two related species, *C. gigas* (Stewart et al., 2014) and *D. reticulatum* (Ahn et al., 2017). For each putatively orthologous gene, the coding regions were aligned by ClustalW with a manual check to correct potential errors. Synonymous substitution rates (Ks) and non-synonymous substitution rates (Ka) were calculated by KaKs_Calculator software with the YN model (Zhang et al., 2006). Genes with Ka/Ks > 1 were considered under strong positive selection, and those with 0.5 < Ka/Ks ≤ 1 were considered as candidates that may have experienced moderate positive selection.

### Peptide Isolation and LC-MS/MS Analysis

The scallop neuropeptides were isolated from the nerve ganglia following the boiling extraction procedures reported previously (Dowell et al., 2006). The samples were then analyzed using Easy-nLC nanoflow HPLC system connected to Orbitrap Elite mass spectrometer (Thermo Fisher Scientific, San Jose, CA, United States). A total of 1 μg sample was loaded onto Thermo Scientific EASY column (two columns) at a flow rate of 150 nL/min. The sequential separation of peptides on Thermo Scientific EASY trap column (100 μm × 2 cm, 5 μm, 100 Å, C18) and analytical column (75 μm × 25 cm, 5 μm, 100 Å, C18) was accomplished using a segmented 1 h gradient from Solvent A (0.1% formic acid in water) to 50% Solvent B (0.1% formic acid in 100% ACN) for 50 min, followed by 50–100% Solvent B for 4 min and then 100% Solvent B for 6 min. The column was re-equilibrated to its initial highly aqueous solvent composition before each analysis.

The mass spectrometer was operated in positive ion mode, and MS spectra were acquired over a range of 300–1,800 m/z. The resolving powers of the MS scan and MS/MS scan at 200 m/z for the Orbitrap Elite were set as 70,000 and 17,500, respectively. The top 10 most intense signals in the acquired MS spectra were selected for further MS/MS analysis. The isolation window was 2 m/z, and ions were fragmented through higher energy collisional dissociation with normalized collision energies of 27 eV. The maximum ion injection times were set at 10 ms for the survey scan and 60 ms for the MS/MS scans, and the automatic gain control target values were set to 3e6 for full scan modes and 5e4 for MS/MS. The dynamic exclusion duration was 30 s.

The raw files were transformed to MGF format by software Proteomics Tools 3.1.6 (Sheng et al., 2015) and then search for the fragmentation spectra was performed using the MASCOT 2.2 (Perkins et al., 1999) search engine embedded in Proteome Discoverer against the translated nerve ganglia transcriptome database. The following search parameters were used: monoisotopic mass, trypsin as the cleavage enzyme, two missed cleavages, peptide charges of 2+, 3+, and 4+, carbamidomethylation of cysteine as fixed modifications, and the oxidation of methionine, acetyl (N-term), amidated (C-term), dioxidation (M), Gln->pyro-Glu (N-term Q), Glu->pyro-Glu (N-term E) were specified as variable modifications. The mass tolerance was set to 10 ppm for precursor ions and to 0.05 Da for the fragment ions. The search result of mascot was exported using the Buildsummary (Sheng et al., 2012) of software Proteomics Tools 3.1.6. The mascot data were filtered according to a significance threshold of Mascot score >20.

## Results and Discussion

### Ganglia Transcriptome Sequencing and Assembly

To enable a thorough identification of neuropeptides, ganglia transcriptome was sequenced and *de novo* assembly was performed. The transcriptome sequencing produced 17,273,790 raw paired-end sequences. After the quality filtering step, 16,542,944 (95.77%) HQ paired-end reads were obtained and used for *de novo* assembly. Finally, the scallop ganglia transcriptome was assembled into 155,937 transcripts of 124,501 trinity “gene,” with an average length of 735 bp and N50 of 1,782 bp. About 33.38% of the transcripts were no less than 500 bp in length, and 19.39 % were at least 1 kb. The longest isoform was selected as the representative for each “gene.” The average length and N50 of the unigenes were 531 and 1,030 bp. Among the unigenes, 27,897 (22.41%) were at least 500 bp, and 11,960 (9.60%) were no less than 1 kb. The nerve ganglia transcriptome assembly not only serves as a reference database for the LC-MS/MS analysis but also provides valuable resources for predicting neuropeptide genes using NpSearch.

### Neuropeptide Precursors Identification

**FIGURE 1:**
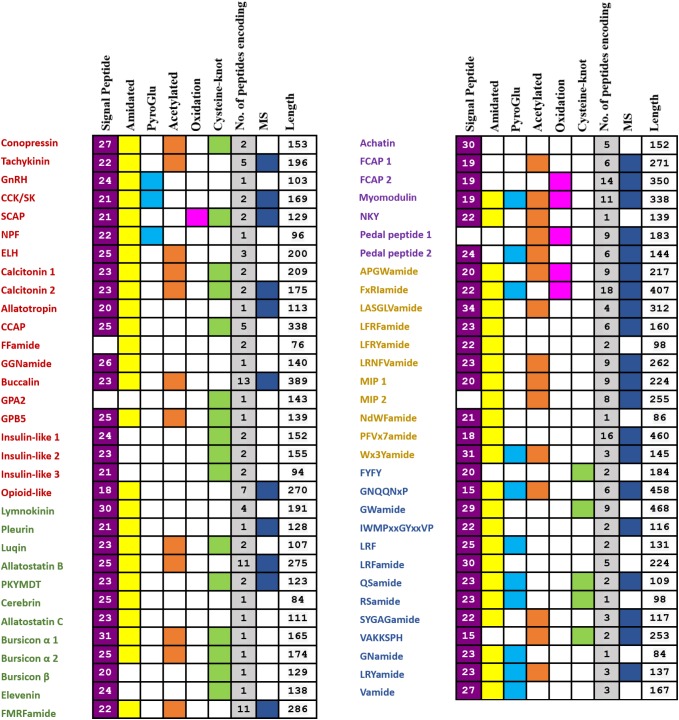
Summary of identified genes encoding putative full- or partial-length neuropeptide precursors from the *P. yessoensis* nerve ganglia transcriptome and proteome. The 20 ancestral bilaterian neuropeptide precursors are in red; the 12 neuropeptide precursors which exist in all the major groups of protostomes are in green; the 7 neuropeptide precursors only found in Mollusca, Annelida, and Nematoda are in purple; the 11 neuropeptide precursors that were only characterized in Lophotrochozoa are in yellow; the 13 potentially novel neuropeptide precursors are in blue.

Based on BLAST and *de novo* prediction, 48 and 60 neuropeptide precursors were identified, respectively, resulting in a total of 63 genes (**Figure 1**). Among them, 50 have been identified previously in other species, and the remaining 13 are potentially novel. LC-MS/MS confirmed peptides from 31 (49.21%) neuropeptide precursors, including 25 previously identified and 6 novel ones (**Figure 1**). Detailed information on the 63 neuropeptide precursors is shown in the **Supplementary Figure S1** and **Supplementary Table S1**, and the peptide information from LC-MS/MS is displayed in the **Supplementary Table S2**. The 50 genes that encode known neuropeptide precursors can be categorized into two groups: (1) 20 of them representing 17 neuropeptides commonly exist in bilaterians, including the 13 families that have been reported before (Mirabeau and Joly, 2013) (Conopressin, Tachykinin, GnRH, CCK/SK, SCAP, NPF, ELH, Calcitonin, Allatotropin, crustacean cardioactive peptide (CCAP), FFaminde, GGNamide, Buccalin), as well as GPA2, GPB5, insulin-like peptides and opioid-like peptide; (2) the remaining 30 genes encode neuropeptides that are only characterized in protostomes, including 12 that are present in all the major groups of protostomes, 7 that are found in molluscs, annelids and nematodes, and 11 that are only characterized in Lophotrochozoa.

Below we will characterize in detail glycoprotein hormones, insulin-like peptides, allatostatin family, RFamide family, and some neuropeptides in relation to mollusc reproduction, cardioactivity or feeding behavior.

#### Glycoprotein Hormones

##### Bursicon α and bursicon β

**FIGURE 2:**
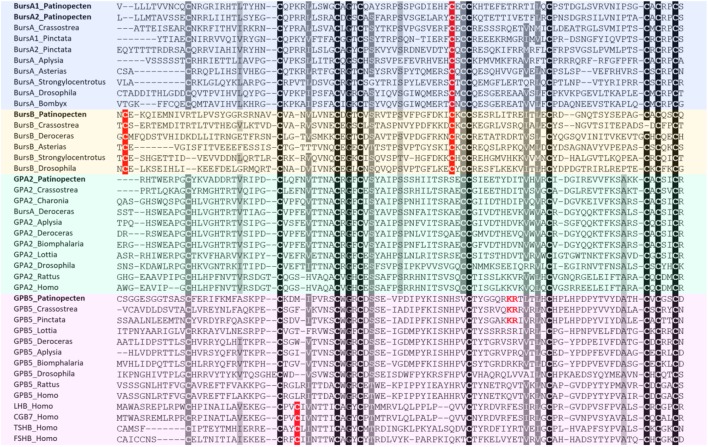
Alignment of glycoprotein hormone precursors. Conserved amino acid residues are highlighted in black, conservative replacements in gray, and other cysteine residues specifically conserved within bus icons in red. The information of sequences used in the figure is displayed in the **Supplementary Table S3**.

Bursicon was first identified in 1965 as a peptide neurohormone in insects. It belongs to a cystine-knot protein composed of two subunits: bursicon α and β (Honegger et al., 2008). A role of bursicons in regulation of cuticle hardening and ecdysis has been demonstrated in crustaceans (Chung et al., 2012; Webster et al., 2013), but little is known about the function of bursicons in molluscs. Till now, genes encoding both α and β subunits have been found in several molluscs, such as *D. reticulatum* (Ahn et al., 2017), *P. fucata*, and *C. gigas* (Stewart et al., 2014). In *P. yessoensis*, genes encoding bursicon were also identified, with two genes encoding bursicon α and one encoding bursicon β. This is similar to pearl oyster *P. fucata* but different from Pacific oyster *C. gigas* and other gastropods (Ahn et al., 2017), suggesting possible occurrence of gene duplication for bursicon α in some bivalves. Sequence alignment analysis shows that most bursicon genes contain 11 cysteine residues in conserved positions, but the first Cys is positioned differently between bursicon α and β (**Figure 2**).

##### GPA2 and GPB5

Glycoprotein family is one group of ancient hormones that consist of two subunits: glycoprotein-α2 (GPA2) and glycoprotein-β5 (GPB5) (Sellami et al., 2011). In vertebrates, GPA2/GPB5 dimer stimulates TSH receptors and is therefore named thyrostimulin (Nakabayashi et al., 2002). The GPA2 and GPB5 have an ancestral bilaterian origin, from which vertebrate LH, FSH, CG, and TSH probably having evolved (Sower et al., 2009). In insects, GPA2/GPB5 participates in ionic and osmotic balance (Paluzzi et al., 2014). Although the GPA2 and GPB5 orthologs have been reported in some molluscs, including gastropods *C. tritonis* (Bose et al., 2017), *D. reticulatum* (Ahn et al., 2017), *A. californica* (GPA2: NP_001191641; GPB5: NP_001191597), *B. glabrata* (GPA2: XP_013094496; GPB5: XP_013088838), *L. gigantea* (GPB5: FC741803), and two bivalves *P. fucata* and *C. gigas* (Stewart et al., 2014), their function is currently unknown.

In Yesso scallop, both GPA2 and GPB5 were identified, which encode precursors of 143 and 139 residues, respectively. Sequence alignment between GPA2 and GPB5 reveals that GPA2 contains 10 conserved cysteine residues, and GPB5 has 9, missing the fifth Cys (**Figure 2**). Besides, there is a unique KR cleavage site in the GPB5 of scallop and oysters, which could be a bivalve-specific feature (**Figure 2**).

#### Insulin-Like Peptides

Molluscan insulin-related peptide is a growth stimulating hormone related peptide involved in glucose metabolism and growth (Ebberink et al., 1989). It may also participate in the regulation of germinal cell proliferation and maturation (Monnier and Bride, 1995). Molluscan insulin-related peptides have been determined in the snail *L. stagnalis* (Smit et al., 1988, 1996; Li et al., 1992b), limpet *L. gigantea* (Veenstra, 2010), and slugs *A. californica* (Floyd et al., 1999) and *D. reticulatum* (Ahn et al., 2017).

In *P. yessoensis*, we identified three insulin-like peptide precursors. Py-ISNL1 is a 152-residue precursor protein comprising a predicted N-terminal signal peptide (24-residue) and two insulin-like domains A and B. The A chain contains five cysteine residues (residues 130, 132, 133, 137, and 146) and B chain contains three (residues 29, 40, and 52), which are likely to form disulfide bridges. Similar to Py-ISNL1, the 94-residue precursor protein Py-ISNL2 is also composed of a predicted N-terminal signal peptide (21-residue) and two insulin-like domains containing eight cysteine residues (A chain: residues 75, 77, 78, 82, and 91; B chain: residues 23, 38, and 50). Py-ISNL3 is a 155-residue precursor protein possessing similar structures, with a 23-residue N-terminal signal peptide and two insulin-like domains that contain three (residues 28, 39, and 51) and five (residues 137, 139, 140, 144, and 153) cysteine residues, respectively.

**FIGURE 3:**
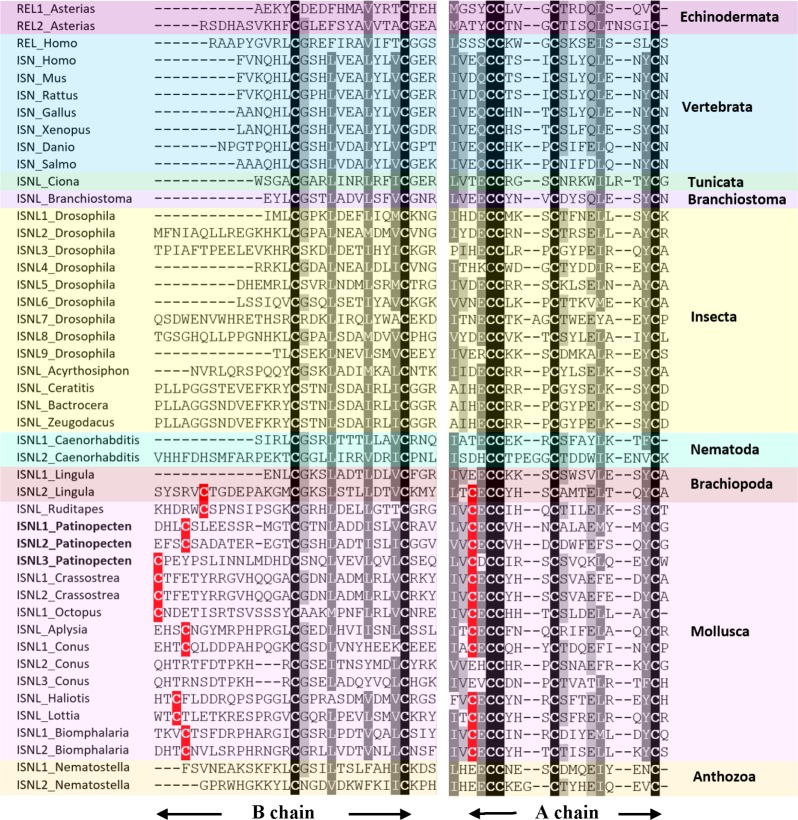
Alignment of A and B chains of insulin-like peptide (ISNL), insulin (ISN), and relaxin (REL) precursors. Conserved amino acid residues are highlighted in black, conservative replacements in gray, and other cysteine residues conserved between Brachiopoda and Mollusca in red. The information of sequences used in the figure is displayed in the **Supplementary Table S3**.

Sequence alignment (**Figure 3**) showed that for most molluscan insulin-like peptides, the cysteine motifs of A chains and B chains are CxCCxxxCxxxxxxxxC and Cx_(9-14)_CxxxxxxxxxxxC, respectively. The extra cysteine residue in both A and B chains suggests most molluscan insulin-like peptides may form four disulfide bridges instead of three as in insects and vertebrates. Similar structure also exists in one insulin-like peptide from *Lingula anatina*, indicating this could be a feature of lophotrochozoan insulin-like peptides.

#### Allatostatin Family

Allatostatins were originally found in insects. They inhibit biosynthesis of juvenile hormone, reduce food intake and appear to be myoinhibitory on visceral muscle in many insects (Nässel, 2002; Stay and Tobe, 2007). Insect allatostatin family consists of allatostatin A, allatostatin B, and allatostatin C with structurally diverse peptides (Nässel, 2002). All three allatostatin homologs have been reported in molluscs but some of them are in different names, with allatostatin A being called buccalin, and allatostatin B also called WWamide. In *P. yessoensis*, all three genes are identified, of which two (buccalin and allatostatin-B) were confirmed by MS data.

##### Allatostatin A or buccalin

**FIGURE 4:**
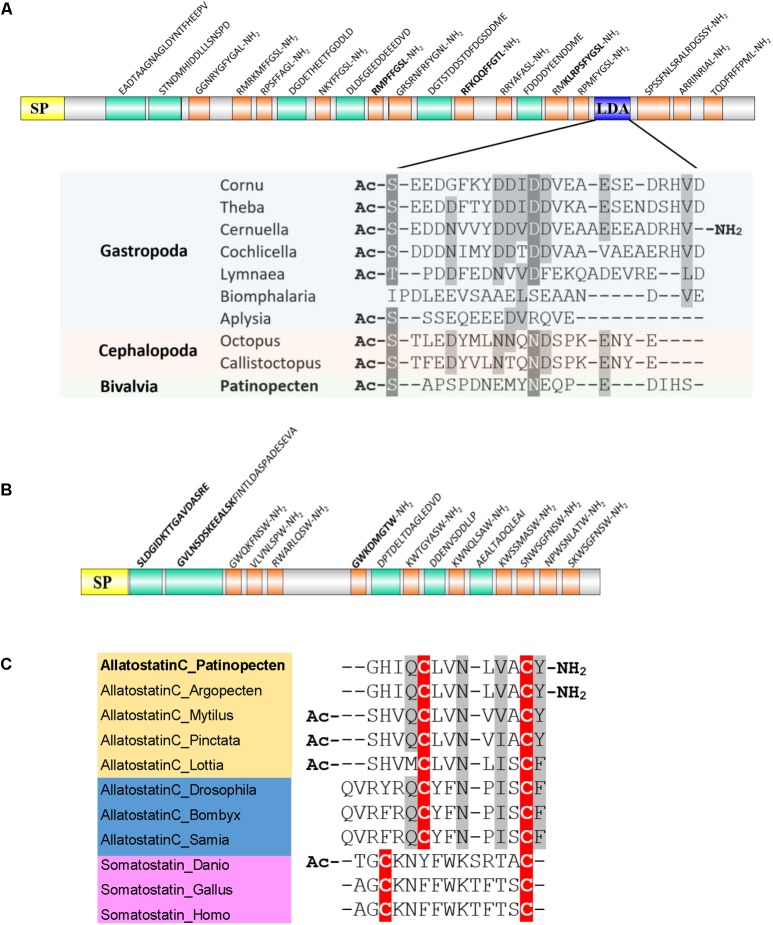
Schematic representation of scallop allatostatin family and alignment of potential bioactive peptides. **(A)** Schematic representation of allatostatin A/buccalin precursor and sequence alignment of LDAs among molluscs. Yellow, signal peptide (SP); orange, amidated peptides; green, non-amidated predicted peptides; blue, LDA. The biologically active peptides confirmed by MS are indicated in bold. **(B)** Schematic representation of allatostatin B precursor. **(C)** Sequence alignment of bioactive allatostatin C and somatostatin. Cysteine residues are highlighted in red, and conservative replacements in gray. All the information of sequences used in the figure is displayed in the **Supplementary Table S3**.

Allatostatin A is a kind of Lamide with a C-terminal FGLamide in insects and GxLamide in molluscs. Molluscan buccalin has been reported in *A. californica* (Miller et al., 1993), *L. gigantea* (Veenstra, 2010), *D. reticulatum* (Ahn et al., 2017), *P. fucata*, and *C. gigas* (Stewart et al., 2014). The *P. yessoensis* buccalin precursor encodes a 23-residue signal peptide and 13 diverse buccalin-like peptides with C-terminal Lamide (**Figure 4A**). Among them, five are GSLamides, and MS analysis confirmed three peptides, including RMPFFGSLamide, RFKQQFFGTLamide, and KLRPSFYGSLamide.

A recent study shows that in the buccalin precursor of helicid snail embeds a love dart allohormone (LDA), which has the function of stimulating copulatory canal contractility (Stewart et al., 2016). Although copulatory canal does not exist in scallops, we found the LDA-like peptide sequence also exists in the Py-buccalin precursors (**Figure 4A**). Moreover, LDA-like peptide seems to exist across multiple classes of Mollusca, implying that LDA may have conserved functions in molluscs. Whether LDA is mollusc specific or coexists in other phyla remains to be explored.

##### Allatostatin B or WWamide

Allatostatin B is also called WWamide due to the existence of an N-terminal Trp and C-terminal Trp-amide. The two Trp residues are usually separated by six and occasionally seven amino acid residues in insects, but only four or five residues in molluscs. Molluscan allatostatin B has been reported in *L. gigantea* (Veenstra, 2010), *D. reticulatum* (Ahn et al., 2017), *P. fucata*, and *C. gigas* (Stewart et al., 2014). In scallop *P. yessoensis*, allatostatin B precursor was also identified, which encodes a 25-residue signal peptide followed by 10 allatostatin B-like peptides (**Figure 4B**). One peptide GWKDMGTWamide was confirmed by MS.

##### Allatostatin C

The somatostatin homolog allatostatin C, originally identified from an insect *Manduca sexta*, is present in a broad range of invertebrates including Arthropoda (Dickinson et al., 2009; Veenstra, 2009), Annelida (Veenstra, 2011), and Mollusca (Veenstra, 2010; Stewart et al., 2014; Ahn et al., 2017). It is characterized by a conserved domain containing two Cys residues and six residues in between. The molluscan allatostatin C has been identified in *L. gigantea* (Veenstra, 2010), *D. reticulatum* (Ahn et al., 2017), *P. fucata* and *C. gigas* (Stewart et al., 2014). The scallop Py-allatostatin C precursor gene encodes a 28-residue signal peptide and an allatostatin C peptide (GHIQCLVNLVACYamide). Sequence alignment of the bioactive peptides (**Figure 4C**) revealed that: (1) both vertebrate somatostatins and invertebrate allatostatins C have two conserved Cys residues, but allatostatin C has an extra Tyr/Phe residue after the second Cys; (2) molluscan allatostatins C peptides are more similar to insect allatostatins C than vertebrate somatostatins; (3) scallop *P. yessoensis* and *A. irradians* share the same allatostatin C sequence, which has an amidated C-terminal Tyr that is different from other bivalves.

#### RFamide Neuropeptide Family

RFamide neuropeptide family is composed of neuropeptides with a C-terminal RFamide motif that is presumed to be an ancient and convergent feature of neuropeptide evolution (Jékely, 2013; Elphick and Mirabeau, 2014). The RFamide neuropeptides display a complex spatiotemporal pattern of expression in the central and peripheral nervous system controlling various biological and physiological processes including cardiovascular regulation, osmoregulation, reproduction, digestion, and feeding behavior (Zatylny-Gaudin and Favrel, 2014). RFamide-type neuropeptides distribute in both vertebrates and invertebrates, but difference exists regarding to the members. In vertebrates, there are five families of RFamide: gonadotropin-inhibitory hormone (GnIH), neuropeptide FF (NPFF), pyroglutamylated RFamide peptide (QRFP), prolactin-releasing peptide (PrRP), and Kisspeptin (Elphick and Mirabeau, 2014). While in molluscs, RFamides only include five genes: FMRFamide-related peptide, LFRFamide, luqin, neuropeptide F (NPF), and cholecystokinin/sulfakinin (CCK/SK) (Zatylny-Gaudin and Favrel, 2014). Some of them such as luqins have been lost in the vertebrate lineage.

##### FMRFamide and FMRFamide-related peptides (FaRPs)

The tetrapeptide FMRFamide was first discovered in the clam *Macrocallista nimbosa* due to its cardioexcitatory activity (Price and Greenberg, 1977). It has also been found in insects (Nässel, 2002) and annelids (Veenstra, 2011), and is conserved throughout these phyla. In comparison to other neuropeptides, FMRFamides are relatively well studied in molluscs. It has been identified and/or functionally characterized in gastropods (Taussig and Scheller, 1986; Santama et al., 1995a; Veenstra, 2010; Ahn et al., 2017), bivalves (Price and Greenberg, 1977; Favrel et al., 1998; Stewart et al., 2014) and cephalopods (Loi and Tublitz, 1997; Wollesen et al., 2010). Except for its cardioexcitatory activity, molluscan FMRFamide also participates in reproduction regulation, precisely reducing the reproductive activities (Brussaard et al., 1988; Di Cristo et al., 2002, 2003; Morishita et al., 2010).

**FIGURE 5:**
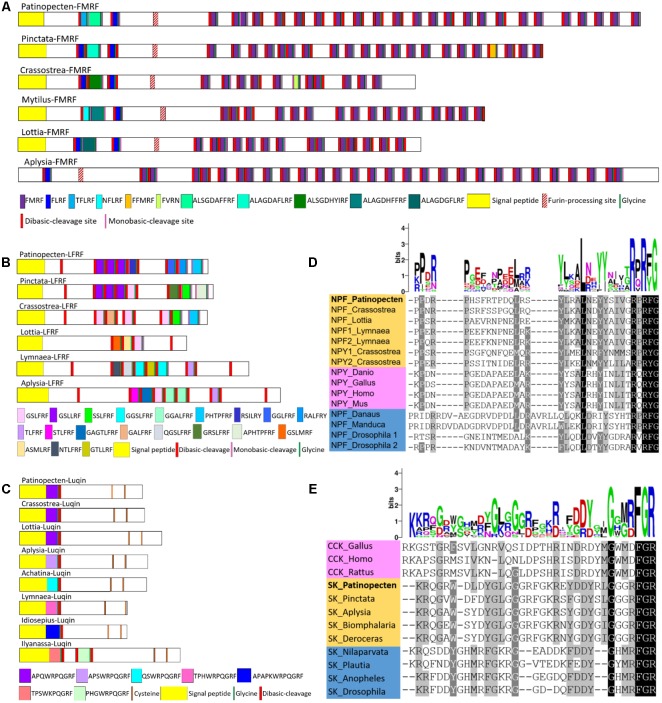
Comparison of the linear schematic organization of **(A)** FMRF, **(B)** LFRF, and **(C)** luqin and alignment of known orthologous peptides from **(D)** NPF/NPY and **(E)** CCK/SK. The height of each letter of the logo is proportional to the observed frequency of the corresponding amino acid in the alignment column. All the information of sequences used in the figure is displayed in the **Supplementary Table S3**.

The molluscan FMRFamide precursors share a common structure, with a tetrabasic furin-processing site (RKRR) that separates the precursor into two domains: the N-terminal region encoding two tetrapeptide or pentapeptide (FLRFamide or xFLRFamide) and a decapeptide (ALxGDxFxRFamide), and the C-terminal domain encoding the FMRFamides. Similarly, the Py-FMRFamide gene encodes a precursor containing a 22-residue signal peptide followed by the two domains (**Figure 5A**). The N-terminal domain comprises a pentapeptide TFLRF and a tetrapeptide FLRF separated by an MS confirmed ALSGDAFFRFamide, and the C-terminal domain contains 24 copies of FMRFamides.

##### LFRFamide

The first LFRFamide peptide was isolated from the gastropod *Fusinus ferrugineus*, where two heptapeptides (GSLFRFamide and SSLFRFamide) showed inhibitory activity on F2 neurons (Kuroki et al., 1993). Since then, LFRFamide has been found to widely exist in other gastropods (Cropper et al., 1994; Hoek et al., 2005; Veenstra, 2010; Ahn et al., 2017; Bose et al., 2017), cephalopod (Zatylny-Gaudin et al., 2010), and bivalves (Stewart et al., 2014), but has not yet been reported in scallops. Here, we identified an LFRFamide precursor gene in *P. yessoensis*, which encodes a 23-residue signal peptide followed by three GSLLRFamides and single copy of RSILRYamide, GGLFRFamide, RALFRYamide, and PHTPFRFamide (**Figure 5B**).

Despite its wide existence in molluscs, study on the function of LFRFamide is limited. There is only one report in *L. stagnalis* showing that LFRFamide peptides can inhibit growth or reproduction, and could be involved in the suppression of host metabolism and reproduction during parasitation by schistosome (Hoek et al., 2005). Further research is required to determine whether the inhibitory function of LFRFamide on growth or reproduction commonly exists in other molluscs.

##### Luqin

Luqin was first isolated from the mollusc *A. californica* and named luqin because it was expressed in the dorsal left upper quadrant (LUQ) cells of the abdominal ganglion (Shyamala et al., 1986; Aloyz and Desgroseillers, 1995). Luqin functions as a myoactive or cardioactive peptide in the African giant snail *A. fulica* (Fujimoto et al., 1990), thus it is also called cardioexcitatory peptide in some species (Tensen et al., 1998; Satake et al., 1999a). Luqins distribute in molluscs and annelids (Veenstra, 2011), but are lost in vertebrates. The molluscan luqin has been reported in *A. californica* (Shyamala et al., 1986; Aloyz and Desgroseillers, 1995), *L. stagnalis* (Tensen et al., 1998), *Achatina fulica* (Satake et al., 1999a), *L. gigantea* (Veenstra, 2010), *C. gigas* (Stewart et al., 2014), and *D. reticulatum* (Ahn et al., 2017). In *P. yessoensis*, a precursor was predicted, which has a luqin peptide (APQWRPQGRFamide), following immediately after a 23-residue signal peptide. The sequences of luqin are strongly conserved among *P. yessoensis, C. gigas* and *L. gigantea*, and all the molluscan luqins have a WRPQGRFamide motif and two conserved Cys residues at C-termini (**Figure 5C**).

##### NPF

NPF is a long neuropeptide consisting of 36-40 residues, and invertebrate NPF is similar to vertebrate neuropeptide Y (NPY). NPFs have been identified in molluscs (Leung et al., 1992; Rajpara et al., 1992; De Jong-Brink et al., 1999; Veenstra, 2010; Stewart et al., 2014) and insects (Nässel, 2002; Liu et al., 2013) and are characterized by a C-terminal GRPRFamide. Due to the structural similarity to vertebrate NPY, some molluscan NPFs are also called NPY, even though their C-terminal sequence ends in Arg-Phe-amide. Molluscan NPF has been reported in *A. californica* (Rajpara et al., 1992), *L. stagnalis* (De Jong-Brink et al., 1999), *H. aspersa* (Leung et al., 1992), *L. gigantea* (Veenstra, 2010), *D. reticulatum* (Ahn et al., 2017), *P. fucata* (Stewart et al., 2014), and *C. gigas* (Stewart et al., 2014), and NPY with a C-terminal RxRYamide has been found in *P. fucata* and *C. gigas* (Stewart et al., 2014). NPF/NPY plays roles in feeding, metabolism, reproduction and stress responses in both invertebrates and mammals (Nässel and Wegener, 2011), and is supposed to participate in regulating energy flows in mollusc *L. stagnalis* (De Jong-Brink et al., 2001). In *P. yessoensis*, only an NPF precursor was predicted, which has a 22-residue signal peptide and a 39-residue NPF-like peptide (QEAMLEPPDRPHSFRTPDQLRSYLRALNEYYSIVGRPRFa- mide). Alignment of molluscan NPF/NPY sequences reveals the conserved RPRF/RPRY-amide at C-termini (**Figure 5D**).

##### CCK/SK

CCK/SK represents an ancestral bilaterian peptide family that participates in digestion and feeding behavior (Schoofs and Nachman, 2006). Vertebrate CCKs possess a common C-terminal motif GWMDFamide (Dimaline and Dockray, 1994; Schoofs and Nachman, 2006; Rehfeld et al., 2007), and insect SKs share a C-terminal RFamide motif (Nachman et al., 1986; Baldwin et al., 2010). The molluscan CCK/SK have been reported in *D. reticulatum* (Ahn et al., 2017), *L. gigantea* (Mirabeau and Joly, 2013), *Haliotis diversicolor* (Mirabeau and Joly, 2013), *A. californica* (Mirabeau and Joly, 2013), *P. fucata* and *C. gigas* (Stewart et al., 2014). The *P. yessoensis* CCK/SK precursor has a 21-residue signal peptide, a MS-confirmed pyroglutamylated QGRWDLDYGLGGGRFamide, and an RFamide peptide (EYDDYRLGGGRFamide). Sequence alignment shows that the two RFamide peptides are highly conserved in molluscs, with a C-terminal motif DYGLGGGRFamide and GGGRFamide, respectively (**Figure 5E**). The molluscan and arthropod SKs share a conserved motif DYxxGxxRF, but arthropod SKs lack the two amino acids between Y and G residues (**Figure 5E**).

#### Neuropeptides With Known Functions

##### Reproduction-related neuropeptides

In molluscs, some neuropeptides have been found to be involved in reproduction control, such as RFamides (FMRFamides and LFRFamides), APGWamide, egg-laying hormone (ELH), gonadotropin-releasing hormone (GnRH), myomodulin, and FxRIamide. We have identified the precursors of all these neuropeptides in *P. yessoensis*. Below we will describe them in detail except for the two RFamides which have been characterized in Section “RFamide Neuropeptide Family.”

###### APGWamide

**FIGURE 6:**
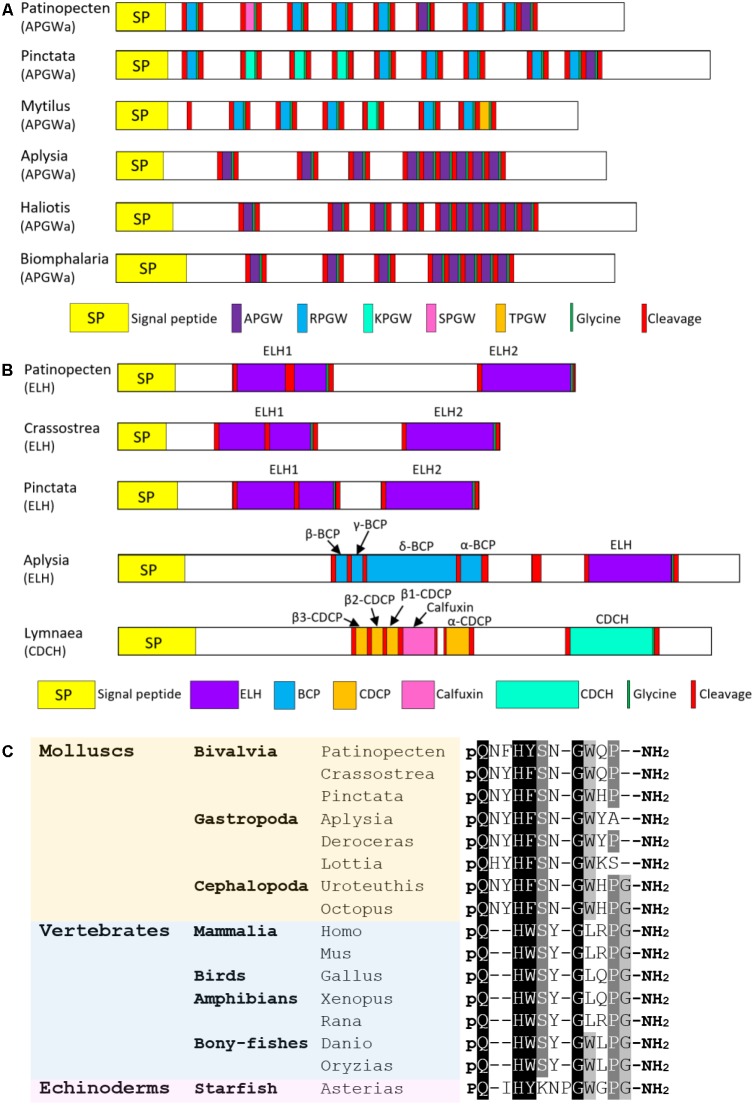
Comparison of the linear schematic organization of **(A)** APGWamide and **(B)** ELH; **(C)** alignment of GnRH peptides among molluscs, vertebrates, and echinoderms. All the information of sequences used in the figure is displayed in the **Supplementary Table S3**.

APGWamide was originally identified in a gastropod *F. ferrugineus* (Kuroki et al., 1990). Till now, it has been found in other molluscs including *A. californica* (Fan et al., 1997), *L. stagnalis* (Smit et al., 1992), *M. edulis* (Favrel and Mathieu, 1996), *L. gigantea* (Veenstra, 2010), *C. tritonis* (Bose et al., 2017), *D. reticulatum* (Ahn et al., 2017), *P. fucata*, and *C. gigas* (Stewart et al., 2014). APGWamide regulates the male reproductive behavior in gastropods (De Lange et al., 1998; Koene, 2010) and has pheromonal actions in bivalves (Bernay et al., 2006) and cephalopods (Di Cristo et al., 2005; Di Cristo, 2013). In *P. yessoensis*, an APGWamide precursor was identified, which comprises a 20-residue signal peptide, six copies of RPGWamide, two copies of APGWamide and one copy of SPGWamide (**Figure 6A**). Interestingly, gastropods and cephalopods only have APGWamide, while bivalve APGWamides contain numerous tetrapeptide repeats that vary in the first amino acid (APGWamide, RPGWamide, TPGWamide, and KPGWamide). The various tetrapeptides seem to have different functions: APGWamide can regulate male reproduction (De Boer et al., 1997) and induce imposex (Oberdörster and McClellan-Green, 2000) in gastropods, and is detected in the seminal fluid in the seminal duct of oyster *C. gigas* (Bernay et al., 2006), suggesting it may participate in reproduction regulation; while RPGWamide, TPGWamide, and KPGWamide can regulate the locomotion of muscle in bivalves (Henry et al., 2000). Whether the three kinds of tetrapeptides observed in Py-APGWamide precursor have diverse functions remains to be investigated.

###### ELH

ELH is a neuropeptide hormone that was first reported to stimulate ovulation in gastropod *A. californica* (Nuurai et al., 2010). *Aplysia* ELH resembles another peptide hormone caudodorsal cell hormone (CDCH) in *L. stagnalis* in both amino acid sequence and function (Morishita, 2017). ELH/CDCH has been discovered in *A. californica* (Strumwasser et al., 1987), *L. stagnalis* (Li et al., 1992a), *A. parvula* (Nambu and Scheller, 1986), *L. gigantea* (Veenstra, 2010), *P. fucata* (Stewart et al., 2014), *C. gigas* (Stewart et al., 2014), and *C. tritonis* (Bose et al., 2017). In *P. yessoensis*, an ELH precursor was found, which contains a 25-residue signal peptide and two ELH-like domains (**Figure 6B**). The Py-ELH1 contains 39 amino acid, about twice the length of Py-ELH2 (20-residue). Schematic representations show that the organization of Py-ELH precursor is similar to that of ELH from oysters *P. fucata* and *C. gigas*, with duplicated ELH-like peptides on the precursors. It indicates that bivalve ELH precursor is different from precursors of ELH in *A. californica* and CDCH in *L. stagnalis*, which also have other bioactive peptides, such as bag cell peptides (BCPs), caudodorsal cell peptides (CDCPs) and calfluxin. Considering that egg-laying behaviors in *A. californica* and *L. stagnalis* are induced through the coordination of various peptides from the same precursor (Morishita, 2017), deficiency of BCPs or CDCPs in bivalves may be related to the less complicated egg-laying behaviors.

###### GnRH

GnRH is a neurohormone central to the regulation of reproductive functions in vertebrates. In molluscs, GnRH has been identified in *O. vulgaris* (Iwakoshi et al., 2002; Iwakoshi-Ukena et al., 2004), *A. californica* (Zhang et al., 2008; Jung et al., 2014), *L. gigantea* (Veenstra, 2010), *P. fucata* (Stewart et al., 2014), *C. gigas* (Stewart et al., 2014), and *H. asinine* (Nuurai et al., 2014). Herein, a *P. yessoensis* gene encoding GnRH precursor was identified. It contains a 24-residue signal peptide followed by an 11-mer GnRH-like peptide (QNFHYSNGWQP-amide) that shares high sequence similarity with other molluscan GnRH (**Figure 6C**). The function of GnRH has been widely studied in various molluscs, but it varies among species. In octopus, GnRH can induce the gonadal maturation and oviposition (Iwakoshi-Ukena et al., 2004; Minakata et al., 2009). It is also involved in feeding, movement and memory (Iwakoshi-Ukena et al., 2004; Kanda et al., 2006). In *Aplysia*, GnRH seems to regulate behaviors, but fails to induce gonadal maturation (Tsai et al., 2010; Sun and Tsai, 2011). In scallop *P. yessoensis*, GnRH can cause an inhibitory effect on oocyte growth and stimulate spermatogonial proliferation (Nakamura et al., 2007; Nagasawa et al., 2015a). The lack of a solid connection between reproduction and mollusc GnRH suggests that mollusc GnRH may serve as a general neural regulator which may or may not involve reproduction. The conserved role of GnRH as hypothalamic regulator in reproduction in chordates is proposed to be a consequence of neofunctionalization following genomic duplication, which also leads to the formation of a functioning pituitary (Tsai and Zhang, 2008; Roch et al., 2011).

###### Myomodulin

Myomodulin is an innervation messenger of the male sexual system of *L. stagnalis* (De Lange et al., 1998; Koene, 2010). It also shows highest expression levels in several male reproductive organs of *H. aspersa* (Greenberg et al., 1997). Myomodulins have been reported in various molluscs, including *A. californica* (Lopez et al., 1993), *L. stagnalis* (Kellett et al., 1996), *L. gigantea* (Veenstra, 2010), *P. fucata*, and *C. gigas* (Stewart et al., 2014). Here, a myomodulin precursor was predicted in *P. yessoensis*, which comprises a 19-residue signal peptide and multiple copies of myomodulin-like peptides with the conserved motif of xxxMLRLamide. Three of the MLRLamides (GGLSMLRLamide, GMNMLRLamide, and PMSMLRLamide) have been confirmed by MS analysis.

###### FxRIamide

FxRIamide is also called S-Iamide peptide due to its common structure xSSFxRIamide (Kuroki et al., 1993). It may be involved in reproduction regulation in molluscs (Koene, 2010; Morishita et al., 2010). FxRIamides have been described in molluscs *F. ferrugineus* (Kuroki et al., 1993), *L. stagnalis* (El Filali et al., 2006), *L. gigantea* (Veenstra, 2010), *D. reticulatum* (Ahn et al., 2017), *P. fucata*, and *C. gigas* (Stewart et al., 2014). The Py-FxRIa gene encodes a 16-residue signal peptide and 17 xSSFxRI-like peptides, including seven copies of MSSFMRIamide, six copies of LSSFMRIamide and single copy of four other variants of xSSFxRIamides. Among them, three (GLSSFVRIamide, MSSFMRIamide, and IPTSSFMRIamide) were confirmed by MS analysis.

##### Cardioactivity- or feeding-related neuropeptides

###### Tachykinin

Tachykinin peptides are widely distributed in Mollusca and Arthropoda (Nässel, 1999). They participate in muscle contraction and cardiovascular function (Van Loy et al., 2010). Tachykinins have been identified in molluscs *L. gigantea* (Veenstra, 2010), *C. gigas* (Stewart et al., 2014), and *D. reticulatum* (Ahn et al., 2017). In *P. yessoensis*, a precursor encoding tachykinin was found. It has a 22-residue signal peptide and five copies of tachykinin-like peptides, of which three were confirmed by MS analysis.

###### Crustacean cardioactive peptide (CCAP)

Crustacean cardioactive peptide was first isolated from the pericardial organs of the shore crab *Carcinus maenas* and was found to be involved in heartbeat regulation (Stangier et al., 1987). It also exists in molluscs, such as *L. gigantea* (Veenstra, 2010), *C. gigas* (Stewart et al., 2014), and *H. pomatia* (Minakata et al., 1992; Muneoka, 1994). CCAP peptides have two features: (1) two conserved cysteine residues resulting in a predicted disulfide bridge; (2) C-terminal amidation. The *P. yessoensis* CCAP gene encodes a 25-residue signal peptide and three CCAP-like peptides. Similarly, *Aplysia* and *Lottia* CCAP precursors code for three CCAP-like peptides, but *Crassostrea* and *Helix* CCAP precursors code for only two such peptides, indicating the number of CCAP-like peptides in the precursor varies not only between but also within classes.

###### Small cardioactive peptide (sCAP)

The sCAPs (-AFPRM/ Lamide) are a small peptide family controlling feeding and digestion, and perhaps even cardioactivity in molluscs (Candelario-Martinez et al., 1993). sCAPs have been reported in various gastropods (Mahon et al., 1985; Veenstra, 2010), bivalves (Candelario-Martinez et al., 1993; Stewart et al., 2014), and cephalopod (Kanda and Minakata, 2006). In *P. yessoensis*, a sCAP precursor was found, which has a 21-residue signal peptide, two copies of sCAP peptides (APNFLAYPRG-amide and AMFSYPRL-amide) that have been confirmed by MS analysis, and six cysteine residues structurally conserved among molluscs.

###### Feeding circuit activating neuropeptide (FCAP)

Feeding circuit activating neuropeptide (FCAP) was first identified and characterized from the cerebral-buccal connective of *A. californica* (Sweedler et al., 2002). Its action with CP2 likely mediates the feeding behavior of *Aplysia* (Friedman and Weiss, 2010; Dacks and Weiss, 2013). FCAP has been described in other molluscs, including *D. reticulatum* (Ahn et al., 2017), *L. gigantea* (Veenstra, 2010), *P. fucata*, and *C. gigas* (Stewart et al., 2014). In *P. yessoensis*, there are two Py-FCAP genes. Py-FCAP1 encodes a precursor with a 19-residue signal peptide and 21 copies of FCAP-like peptides, of which SLDRLGGAFIHGY, ALDPLGGVYLHGY, SLDPLGGMWIHGY, GLDRLGGAYLHGF, and SLDRLGGAYLHGF were confirmed by MS analysis. Py-FCAP2 encodes a precursor that contains a 19-residue signal peptide and 13 copies of FCAP-like peptides, of which MDRLGSGLI, AIDRIGSGLV, MLDRVGMGLI, LLDRVGMGLI, MLDRMGSGLI, and MLDRLGSGLV were confirmed by MS analysis.

#### Other Neuropeptides

Full and partial-length *P. yessoensis* genes were also identified that encoded neuropeptides with identity to achatin (GFWD, GYGD), allatotropin (GFRQGIVMRIGHGFamide), cerebrin/PDF-like (NAGTIDSLYNLPDLFAAamide), cono-pressin (CFIRNCPPGamide), elevenin (RPKIGRRFCAHYPFAPRCLGVAA), FFamide/SIF-like peptides (ALSRLLGQQPLLFamide, GMNPNMNSLFF), GGNamide (GKCRGRWSIHACLGGNamide), LASGLVamide (MMDPLANGLVamide, FMSSIANGLI, PFGQLANGLIamide, and 11 YxxxSLASGLIamide), LFRYamide (LPFRFamide, LPFRYamide), LRNFVamide (SRELVamide, LRHFIamide, GRYFVamide, ARYFLamide, YRYFLamide, RYYFLamide, RRFFLamide, MRYFLamide, and FARHFLamide), two MIP variants (9 -PxFVamide variants and 8 -PxFVamide variants), NdWFamide (NWYamide), NKY (KVFWQPLGYVPASMRMSPNNKHKASQKDVGRKGFRYami-de), opioid (YGTLFMSRNGamide, YGTLNLGSGRGLRY-RYGRYamide, and YGMLFLGRNKNRGGYRYGSRamide), two pedal peptide variants (25 variants and 6 variants), PFVx7amide (21 variants), PKYMDT (PKYMDT, ELGDMMQELVYNALKELVSKamide, RRHLSYCLRRSGPNFVPYPCYKYGamide) and Wx3Yamide (SNKWSIAYamide, LRQGWNIAYamide, and QQGWHIAYamide) (**Supplementary Figure S1**).

The neuropeptides identified in *P. yessoensis* cover almost all of the molluscan neuropeptides. In addition, we present for the first time the existence of calcitonin/DH31 (TCNIGVNSHFCALADLDSKIRSREWLNSIYSPamide, TCAVEVGGTCRTEWASSIADQYYYLLGPHSPamide; SCKLNLGFHCQTEEYSAIADMYNFLQSAMSPamide, FLNDEAPSCLVSATDCSMGYIDPIEGFIDVVSNPNSPamide), lymnokinin (PNFHPWA amide, AFHAWGamide and DFGAWGamide) and pleurin (IFYTNKEGNDFPRIamide) in a bivalve (**Supplementary Figure S1**). We also found that two novel neuropeptides (Wx3Yamide and PFGx8amide) of oysters (Stewart et al., 2014) are present in the scallop (Wx3Yamide and PFVx7amide).

The 13 newly identified neuropeptides were named FYFY, GNamide, GNQQNxP, GWamide, IWMPxxGYxxVP, LRF, LRFamide, LRYamide, QSamide, RSamide, SYGAGamide, VAKKSPH, and Vamide according to their sequence patterns or peptide features. Six of them (GNQQNxP, IWMPxxGYxxVP, LRYamide, QSamide, SYGAGamide, and VAKKSPH) have been confirmed by MS. Three neuropeptide precursors (GNamide, LRYamide, and Vamide) have no similarity to the sequences in GenBank, suggesting they may represent scallop innovations. The remaining ten show sequence similarities to uncharacterized proteins from lophotrochozoans and/or ecdysozoans. In addition, we assume two neuropeptides, LRFamide (FQQQLRFamide, PQLRFamide, RFMSHMRFamide, DFQFDYKSLQPQERSamide, and FRPQGRFamide) and LRF (GQDLRKLILKKLRF and QISDSPSVRMPSLRFamide) may be new members of RFamide family.

### Neuropeptides Potentially Related to Scallop Shell Growth or Eye Functioning

In order to identify neuropeptides that may be related to some scallop-specific characteristics, we first compared our data with the recently released scallop genome (Wang et al., 2017). According to the results, 49 of the 63 neuropeptides are annotated in the scallop genome, accounting for 77.78% of the identified neuropeptide precursors. Fourteen genes are not annotated in the genome, possibly because RNA-seq-based evidence was used for gene prediction, but the adult tissues used for RNA-seq library construction does not include nerve ganglia. Therefore, some ganglia-specific genes may not be annotated in the genome annotation.

We then examined the expression levels of the 49 neuropeptide precursors in different adult tissues as reported by Wang et al. (2017). It showed that two neuropeptides (insulin-like peptide 3 and LRYamide) are highly expressed in mantle in comparison to other tissues (**Supplementary Table S4**). Since mantle is the tissue that encloses the animal within the shell, it is widely accepted that mantle plays a vital role in shell formation and growth (Jolly et al., 2004; Takahashi et al., 2012; Joubert et al., 2014). The high expression of insulin-like peptide 3 and LRYamide indicates these two genes may be involved in the regulation of shell growth. Previous studies of insulin-like peptides in snail and oyster support our assumption: insulin-like peptides can stimulate protein synthesis in mantle edge cells, and regulate the growth of the mantle edge and shell (Abdraba and Saleuddin, 2000; Gricourt et al., 2003). The other neuropeptide LRYamide is newly identified, therefore remains to be studied.

We also found four neuropeptide genes (FxRIamide, RSamide, VAKKSPH, and GNQQNxP) exhibited specifically high expression in the eye (**Supplementary Table S4**), suggesting these genes may participate in the functioning of scallop eye. Among them, FxRIamide is the only one that has been reported previously, but it is found to be involved in reproduction regulation (Koene, 2010; Morishita et al., 2010), rather than eye functioning. The other three genes are newly identified in our study. Therefore, it remains to be investigated in terms of whether these neuropeptides locate in similar cell types and what functions they play in the eye.

### Selective Pressure of the Neuropeptides

The selective pressure of the neuropeptide precursors was examined by comparing Ka/Ks ratios among scallop *P. yessoensis* and two related molluscs (**Supplementary Table S5** and **Supplementary Figure S2**). Results showed that there is no neuropeptide gene with Ka/Ks > 1 and most genes are with Ka/Ks < 0.5, suggesting that the neuropeptide genes are under purifying selection. Only two neuropeptide genes (GGNamide and insulin-like peptide 2) exhibited signs of positive selection with 0.5 < Ka/Ks ≤ 1 between *P. yessoensis* and *D. reticulatum*. In comparison, the glycoprotein (GPA2 and GPB5) and RFamide family (CCK/SK, FMRFamide, luqin and NPF) have smaller Ka/Ks ratios, implying they are under stronger purifying selection.

## Conclusion

In this study, we described the neuropeptides of *P. yessoensis* using the transcriptome and proteome of nerve ganglia. Sixty-three genes are identified which code for precursors of 50 known and 13 potentially novel neuropeptides. Although some of the previously identified neuropeptides have been functionally characterized in other molluscs, it remains unknown whether the functions are similar in scallops. Besides, the functions of many known neuropeptides are still unexplored, not to mention those novel ones. Further research is needed regarding when and where these neuropeptides express, what GPCRs they interact with, and what functions they exert. This study paves the way for a complete understanding on the roles of neuropeptides in endocrine regulation of various physiological processes in bivalve molluscs.

## Author Contributions

LZ and ZB conceived and designed the experiments. MZ, WL, RL, and XX performed the experiments. MZ and LZ analyzed the data. SW, ZB, YW, YL, and XH contributed reagents, materials, and analysis tools. MZ and LZ wrote the paper.

## Conflict of Interest Statement

The authors declare that the research was conducted in the absence of any commercial or financial relationships that could be construed as a potential conflict of interest.

## Footnotes

1 http://www.cbs.dtu.dk/services/SignalP/

2 http://www.csbio.sjtu.edu.cn/bioinf/Signal-3L/

3 https://github.com/wurmlab/npsearch

## Supplementary Material

FIGURE S1 Protein sequences of the full-length or partial-length neuropeptide precursors of *Patinopecten yessoensis*. The predicted signal peptides are highlighted in yellow; likely convertase cleavage sites are indicated in red; cysteines are highlighted in pink; C-terminal glycine residues are highlighted in green; likely biologically active neuropeptides are highlighted in purple; peptides found by MS are indicated in blue; biologically active neuropeptides confirmed by MS are indicated in bold.

FIGURE S2 Distribution of Ka/Ks ratios of neuropeptide genes between *P. yessoensis* and two related molluscs, *C. gigas* **(A)** and *D. reticulatum* **(B)**. The solid line shows the threshold of Ka/Ks = 1, the dashed line marked the threshold of Ka/Ks = 0.5, and the short dashed line represented threshold of Ka/Ks = 0.1.

TABLE S1 Detailed information of the identified neuropeptide precursors. In the list are the accession numbers of the protein sequences, the results of *de novo* prediction and homology searching, the gene ID from scallop genome, and the protein annotation.

TABLE S2 List of peptides molecularly characterized by MS analysis of scallop nerve ganglia. Peptide sequence was validated according to a significance threshold of Mascot probability based score >20 and checked manually to confirm the Mascot assignment. Na.a and Ca.a: flanking amino and carboxy amino acids on the precursor.

TABLE S3 Accession numbers for sequences used in **Figures 2–6**.

TABLE S4 The RPKM expression values of the neuropeptides potentially related to scallop shell growth and eye functioning in adult tissues/organs of *P. yessoensis*.

TABLE S5 Detailed information of the Ka/Ks ratios of neuropeptides between *P. yessoensis* and two related molluscs, *C. gigas* and *D. reticulatum*.
