# Supplementary material for: Identification and Characterization of Neuropeptides by Transcriptome and Proteome Analyses in a Bivalve Mollusc Patinopecten yessoensis
Source: Front Genet. 2018 Jun 5;9:197. doi: 10.3389/fgene.2018.00197 (PMC5996578; doi:10.3389/fgene.2018.00197)
Supplement: FIGURE S1 — Protein sequences of the full-length or partial-length neuropeptide precursors of Patinopecten yessoensis. The predicted signal peptides are highlighted in yellow; likely convertase cleavage sites are indicated in red; cysteines are highlighted in pink; C-terminal glycine residues are highlighted in green; likely biologically active neuropeptides are highlighted in purple; peptides found by MS are indicated in blue; biologically active neuropeptides confirmed by MS are indicated in bold. [file Image_1.PDF]

Achatin

MAYINAEVSHWLCTCVVIATMLMVLGRCEAGILDKSDESEINFRSTNIEGREHGYMQESEFKRGFWDKRGYGDKRGYGDKRESFEPEVGSTGLLSYFRSVL
PLVLDTYAENVAMTKDKQLHQNDLKRGFWDKRGYGDKKRSYPHLSGRYLKF*

Allatostatin B or WWamide

MKFQMSITRLLCLTLLVLKTAYCMAKSLDGIDKTTGAVDASREKRGVLNSDSKEEALSKFINTLDASPADESEVAKRGWQKFNSWGKRVLVNLSPWGKRR
WARLQSWGKRSIDPSAIDETGKDDIDSEITPEQALLYNEGDKR**GWKDMGTW**GKRDPTDELTDAGLEDVDKRKWTGYASWGKRDDENVSDDLLPKRKW
NQLSAWGKRAEALTADQLEAIKRKWSSMASWGKRSNWSGFNSWGKRNPWSNLATWGKRSKWSGFNSWGKRNAADDLEQ*

Allatostatin C

MIGRDVYRLCFMLGTMFVCTLLITTAKAQSADENLSSALQAGDGEMMDMGGRGGLQDAAYAYRQLALIQDEEDTLLRMMDNLKTKMEHVKVRKRGHIQ
CLVNLVACYGKRK*

Allatotropin

MRTCCCLLVLCFVTVSIIDALPQRTLTRTKR**GFRQGIVMRIGHGF**GKRGDNLFDDFARTTEEENDSQFLMRVEELTEQLADHPEIAEALIRKFVDTNGDGVV
TGEELLGKPEV*

APGWamide

MDSLTVGILSFILTFNVLSASSDTDIMDKRRPGWGKRDGQSEESTIEEFGEPDKRSPGWGKRDSTDFLDNSLDKRRPGWGKRSDILDRIAMLKRRPGWGKRT
VSDFSDLDKRRPGWGKRDSEGIDMEIRAPGWGKRNSIDDREFSLYDKRRPGWGKRSDNIELETRRPGWGKRAPGWGKRSMDVCLSMKEKAEYLINSAIEI
ETEYASLCGIRDSRS*

Buccalin or Allatostatin A

MTNGQHKSQPLTTLFAFLCLVIAVHCSKESLTSRDTKLVNSIQKREADTAAGNAGLDYNTFHEEPVKRSTNDMHIDDLLLSNSPDKRGRGGNRYGFYGALG
KRMDSEEMEKRRMRKMFFGSLGKRRPSFFAGLGKRDGDETHEETFGDDLDKRGRRNKYFFGSLGKRDLDEGEEDDEEEDVDKR**RMPFFGSL**GKRGRSR
NFRFYGNLGKRDGTSTDDSTDFDGSDDMEKR**RFKQQFFGTL**GKREDDLDLEKRRRYAFASLGKRFDDDDYEENDDMEKRRM**KLRPSFYGSL**GKRRPM
FYGSLGKRSAPSPDNEMYNEQPEDIHSRRKRSPSSFNLSRALRDGSSYGRARRINRIALGRRLIRRTQDFRFFPMLGKRSFDTSDGPEDNGEY*

Bursicon α1

MAEMAKFPRIEQICVYLVLLLTVVNCQCNRGRIRHTLTYHNCQPKRLLSWGCAGTCQAYSRPSSTVPGDIEHFCECCKHTEFETRRTILQCPSTDGLSFRRIA
VRVNIPSGCACRPCSALPHHILSAEEELRNGKRTSKNQTVKNVAPFAYNETIEDVDDVDSLK*

Bursicon α2

MANTSNLQRAAQMCISFLLLMTAVSSECNRRLIIHTLSYEGCQPRRLLSRACDGSCASFARPSVDISGELARYCECCKQTETTIVETFLRCPNSDGRSFRRKRILVNIPTACACRPCSALPAYIIAAEDVLQNGKRSPSFDFLSQMNGTVLHEDDGDDSIAERSKLSKVVKGQL*

Bursicon β

MTFSVIATLMLCMFLRGGESLVDNCETKQIEMNIVRTLPVSYGGRSRNAVCVANVLVNECDGTCVSRVTPSVSQFPGFDKICKCCSESRLITREITLTECRDGNQVLENVHLTTSYSEPAGCACQSCQN*

Calcitonin 1

MTNTYVWVCLTCMYTLPSLVLSTEEPQKQITPGARLVRSLDEARHDINGMTNTISSLDEELASKQKRTCNIGVNSHFCALADLDSKIRSREWLNSIYSPGKRSMGDTQKQEDDMDEVTRELKELLQKRINLAKLKELLRDAEQDIVQQRKRTCAVEVGGTCRTEWASSIADQYYYLLGPHSPGRRRRRSPIRLFRRKIATQKLFGKPVM*

Calcitonin 2

MVDACACLIFASFLGLFASAHAQDLHKGTRALVSNLDDVSVELVDAVRAVRRLKKFLNDEAPSCLVSATDCSMGYIDPIEGFIDVVSNPNSPGKRSVRSVENRTLLQKRYVNDILNKRELIGRMQSTLTDILNVIHNERKRSCKLNLGFHCQTEEYSAIADMYNFLQSAMSPGKR*

CCAP

MESSTLNMLWHTVLLILMVAVTCNAGRGLDIDEALQQILSENDRESYYPLDNTDLDNSEQAVSDLIRRLQPEDRLSKRVFCNGFRGCRGGKRTVPMKQQRLLPEEPPVQKRPFCNGFFGCANGKRSIGAPLRQPDPAQDEIETVELRKRLFCNSYGCLNGKRSSLYQTLVERLRNGKLDIGQADSNLPIRSNSDQHPILVTTRKSLRQLRDDDDEDEDVSKFPLNASNAAEWCKNCNLLKLIMPQLYAFRTEETFQPPYDIQLVNAADRMSRELMTSDNLATDDNEDMLLPEESREQIDNFQIDKKCSPMSSFSKACKTSKKSRQRRRLQRKTFKGSP*

CCK/SK

MINQKDSLLALALVLALSACVTYCMPTRLLSDRSAKHVSRLTDILTDLRTLQDKHAAYSDQLNSDLLHKRSDDRKERESFDLLIQLNDPVKIQEVPKIKGDESLLVPDDVISGIDKR**QGRWDLDYGLGGGRF**GKREYDDYRLGGGRFGRDLNHVRDMDHVDLNTFDD*

Cerebrin

MPCRSSLVCLGLVFIIALTFTRGSARSFQASLDQRERQDIMVLAARIIKIAMSASSSSNDVMDKRNAGTIDSLYNLPDLFAAGR*

Conopressin

MEWRLYVSTRLCLTCVLLCVLVLDCQGCFIRNCPPGGKRSMGMMTRATHQCASCGPGMRGQCVGPNTCCGDFGCLMGTEESRVCMKEDDSTEACAVRGSSCGSMGQGNCVSEGLCCDAYACSYNNKCKVNSGKEDREILSLLNRLLHTNDYTD*

Elevenin

MPTITPEYLVLTFMVIFVAGMVNARPKIGRRFCAHYPFAPRCLGVAAKRDGEEANKMANGGLPDYGDIASLVLRSKSDIDDSPVDDWDSHEGTDDVSKAVI
VPLGVLSKILGTKPAAALNKAQKRRLDMWLDYDTGRE*

ELH

MKIQNNHLLLVSLVMIYCTAVNTYALDKTASDILLEQHHSGNSGVNVPFEKRAIPLSLNGDLRMLARMLYASQRRRRVDRFASVRQQMSNLGKRSGGVQH
DKDNDDPIRNSQDTSIRTEQQTQIRSTVPDYRLVSADGYQNVHPRSSLAYEDPEEFQKRSQRLSINGALSSLADMLAASGRRQLKEELAVNRQRLLKLGR*

FCAP 1

MFTKFVIVTIFCWTTLVNCGPVKHDTPVSSTDNQPKRVQRSTPQEIHEQIMNELRGWTPSPADTEVSSGHNNDPLDRLGGTYVHKGTRDADDAYDLLSVVE
EDALPSEGDSTEDNVIPDEEVSFGPSADKFNALRGFLQAIKEGNETPTKR**SLDRLGGAFIHGY**KR**ALDPLGGVYLHGY**KR**SLDPLGGMWIHGYK**KRGL
DPLGGAYLHGFKRSMDDADVDSKRTLDRLGGAYLHGFKRAMDNDDDNSKR**SLDRLGGAYLHGF**KRAMDNDDV

FCAP 2

MTQLSNLLVVLAAVPLAWGQHSFNDIDDDDVGRDKRR**MDRLGSGLI**KRMYDGDADSKR**AIDRIGSGLV**KRKDSVIERLRPTVELLVLQSLMEQRDHQSA
RQDKR**MLDRVGMG**LIKKRPLDQVGTTEKR**LLDRVGMGLI**KKRHLDRIGMGLIKRPVDRVGIKRNPGYKMSTQDIGAPKR**GLDRLGAGLI**KRFGEQLQP
GKR**MLDRMGSGLI**KRADDTDEIHIDEKR**LIDRIGSGLV**KRLSDDESIDQKR**MLDRMGSGLI**KRLSDDSKEEKR**MLDRMGSGLI**KRFNNNDKNAMDKRY
LDRMGSGLIKRMNDDDIETNKRYIDRMGSGLIKRSDANGQVDKR**MLDRLGSGLV**R*

FFamide

MNLKIVCTALVLSTLLLLSHASQSQPSTRALSRLLGQQPLLFGRRGMNPNMNSLFFGKRSMYNSPTNVEDVREALASIYSICQTVMQTKANLVDSEDP*

FMRFamide

MRTLSYVGLLAAVLSMYGGVLGDYLTDWCLDNQPECNGIIAAATGTDEDAKEKRTFLRFGR**ALSGDAFFRF**GRNPVQFEDKRFLRFGRNGGSEGIDELLR
HALNKVESVERATGLKLRRKRSVEPIQPVKNVDEIEEASKQSAEASKADTDKKKRDADDADENKEKRFMRFGRTPDDETGDEDMAKRFMRFGRFMRFGK
SGDEKRFMRFGKKSDTEGDEDKDKRFMRFGKRSDDEMEADKRFMRFGKKSDDEMEADKRFMRFGKRSDDEIGADKRFMRFGKRSDDEIGADKRFMRFG
KRSDDEVGADKRFMRFGKRSDDKMDADKRFMRFGKRSDDEMEADKRFMRFGRAGPEADKRFMRFGRDGADEKRFMRFGKKSDSDNEAEKRFMRFGR
DGADKRFMRFGRDGTDTEADKRFMRFGKKSDETDSDMEENKRFMRFGRDNNQDKRFMRFGRSGADDEKRLMQYGKFDKRFMRFGRSGQNEDKRFMR
FGRSGQLEDKRFMRFGRSGETEDKRFMRFGRSDSSDDSEAAKRFMRFGR*

FxRIamide

MYLVRPWSMLALTLLYFKLTKAESEAENDADADSISHNRVAR**GLSSFVRI**GKDGEDHSVDGPAKRLSSFMRIGKAYDSSKENENDLMDENKRLSSFMRIGK

SSYEPQEDKRLSSFMRIGKSSDEPIDKRLSSFMRIGKSSDEPIDKRLSSFMRIGKSDDGSYAKR**MSSFMRI**GKSLDDEPQDKR**MSSFMRI**GKSLAENAVDKRL
SSFMRIGKNSDEPLDKR**MSSFMRI**GKSDYSDPEAKRKSSFMRIGKGSDESLMKRLSSFMRIGKSMENPEDKR**MSSFMRI**GKSMDNPENKR**MSSFMRI**GKS
MDNPEVKR**MSSFMRI**GKSTGDENEASDVDTQSEEEQPEKR**MSSFMRI**GRSADESGADMKRAFIRIGK**IPTSSFMRI**GRRPYVRVGRLGHSSFIRIGKRADD
SV*

FYFY

MCSTTCWIAVLATFVACTRGTSITRYQHFPLPMCSEDTVCSEISELPSIKEENNYFSWMMCQCPGESVCPKSPGKQTIEVTGSKWYGMCRPKSNIRSCSPGEV
GEEYYMGVREIPFGTFTRVHCLCPLLEYEEGQPEMDASNPKSIYQLICSGTGRLQAAMKRGRGGSRYGGGKNGRRFYFYRK*

GGNamide

MNHRCIWLFAATCCVYLSVNFSSVSAGKCRGRWSIHACLGGNGKRSGPPSALPNSGENSGLFLKKLLLRSEDPVSGSDRYSDMDSFYPPIVQDTEDAMDMP
MVLDNPDLSTEQLQKLSDLVDELIYRKKLRSLSDEAGLV*

GnRH

MSSYTQILVAQLLLAGLLVAVVSGQNFHYSNGWQPGKRAPMMTSGTQLCSFRPHIKALLLWIIEDEVKRIKSCGSSGYDDIINLLQSKQSGLPLSSGMPSDSQ
*

GNQQNxP

MRVLVLLSLLGLALSAPLLEENEAEKKPIVKR**SEEGIGFGNQQNLPK**IKKKSDPAMLADNVGSVLAETPKELTPEDHDEDDAVMQEPDSGNSADSAAKVE
EELNAIIKEEQMAQQVAEDEAGETLPGGDEEPETPIEEHSVSTEGSNGGADTEENTVEVVNPEDVAAAVAAAEEPQQELLLQNNGISPENNEAIKELMQENM
VNSEPVDDGDNAQEQFAEVVDAPDGPERQDNGAEQSLEMLENPLEAYRMYRNYQNFLSTYPNNYADAYRNYQNGRRRRASSTLMSRLRNREIKRNHRIK
RELPYGDSSLFPYFYPEQEQEQEQEDAMYRVLPEQEILGNEPYTLFPEEALEEELAAEEGLPYADEDEEEYGTPVLYDGKMGYFLPSKRQDMLSFVPGNKRE
SYFFPFSKEPETHYKAFVPEKRSYLESYGDLVRLARALAMEPQDREQYLQGYEWE*

GNamide

MNSLPYVTVSMLLLLSHCDLGHAQWAQTFGWGGAGNGKRSGITDPTTCNMDLQLVKLMSTLIQHELKRLKTCSEQKIPQDLLDI*

GPA2

MGTSEILSGPLCKLRRRDVTNFIITIFRIFLVLNTLTGLTYSRHTWERPGCYKVADDRTVRIPDCLQFNVTTNACRGFCVSYAIPSSARTRLRNPSHIITSRSECCS
IEETYDITVHVRCIDGIREVVFKSAKKCACSICRQG*

GPB5

MVSLWGYLPLTLLISFLALCSGSGGESGGTSASCFERIFKMFASKPYRTPSGIELPCKDMIPVRSCWGRCDSSEVPDYRIPYKISNHSVCTYGGQRKRTLTLTH

CHPLHPDPTYTVYDATHCVCGSCDSDFTSCENLNG*

GWamide

MARHFVNRSHLHILVVMLTFHRLQQRVDASPSLRGNNDSTIKKAINFKFPVTIERDTLNRTGRIVNRQEDKRGWEAIELSRRKRDDSITVNWKQRESDRGAT
HWATLNQNDNEAASKRGWNLFTLNKKRGWDGIDFPWGKRSKKENFPKQKWNGVNIAFRKLSNTNKDSQQERGIADNIMTQASSAVSEESQHQTHDSESD
DVSRKRGWDGVDFLFKSHKDPNMRGWGGGDFTTGKRSADREDTKTGWDSAKYSPVNHSDDKESNKRGWDGIDFTFGKRTNNIKSHIKGSNSDDFSTEK
RSNENESHKRGWGGVDVTFGKRSNDYESNKRGWDGVDFPFGRRSDSPYPKQDWAGMDLVLGVGLDTNRVQREKRGWEGLNFHYRVVNGYGPSQSSDY
PKRGWEGVDTCACCRSNRWVLCCLTCNRNDSKPKQRLTTKSYKDMFYVRDTVCKCCYATKVKDCCDLCDVMS*

Insulin-like peptide 1

MYSGIKLAISAIILLSYLSRAMANPDHLCSLEESSRMGTCGTNLADDISLVCRAVYNKREGSQIRDGSWSRYPNIVMRREADRPVVDWNSTAKILDRIKLKNL
AKLGNAALDKRNAFSFISTIRKPGLVCECCVHNCALAEMYMYCGTENNK*

Insulin-like peptide 2

MYGTINLTIAAICIYYLPSALADLEFSCSADATEREGTCGSHLADTISLICGGVFNMPDKRDGPAIRANPWSRFRSIVLGKRDTQRALVDNNEDWKDTVKAL
DKIKRKNIAKLKKVSLTKRNAFQYISTLRVSGVVCECCVHDCDWFEFSQYCGK*

Insulin-like peptide 3

MTLMSITIFIYVICCTAIYGHRCPEYPSLINNLMDHDCSNQLVEVLQVLCSEQSTGTVGSHGRVKRDQAMTNLVCDCCIRSCSVQKLQEYCWLT*

IWMPxxGYxxVP

MNSQNLLQCVIMFLSALTISLALKEAMATNDDVDAPLSADNIGYIIGKLQRRSGLSLLAPPSVYGSKFAPYLQKTPDKRARYLIWMPAQGYVSVPQEDISNG
GGGSPSSSKVFRYG*

LASGLVamide

MTASTLRSNMDSLLSTLGKCTLVILYITGGWCGTQDVSHDDGSLTQEDKRGLDSIANSLDLASGLEEKRMMDPLANGLVGKRYMDSLASGLIGKRFYDDSA
SDLIEKRYIGGIANGLIGKRYMGSIANGLIGKRYMGSIANGLIGKRYMGSIANGLIGKRYIGGIANGLIGKRYIGGIANGLIGKRYMGSIANGLIGKRFMSSIAN
GLIGKRYVDNIASDLIGKREDNGEMHEEKRYIDSLANGLIGKRSDDEEGYDNERSVVDKRYIDNLASGLIGKRSNSYRHHDLQALLDKR**PFGQLANGLI**GK
RSGDD*

LFRFamide

MDTKAMVICGAALFLAIIDLAFTDDGKLVDSDQAEIKKRMAPEIAYEGYNPYADLSDEDLAEVIKRGSLLRFGKRGSLLRFGKRGSLLRFGKRRSILRYGKR
FDDQDENMELPLEDYADAGDDDKRGGLFRFGKRRALFRYGRSADKPHTPFRFGRREEEY*

LFRYamide

MTTLQSLCVVALLLCLCEHTVARSIECSTLCTQGYSITSCECWRFKVKLPFRFGKRGRLPFRYGKRDSPVISSYDSSIEKPSYEDALDLLRSFSSDDY*

LRNFVamide

MYRVSQSACLLLYLAQLVILAQCIDLSGEFSSHTSSSNRGKRETDDDFEDDVAKRSRELVGKRSDGEGLTGKSTLDKRLMPYDELEKRLRHFIGKRTDDEQP
FEKKSRSRGRYFVGKREDDVEDEEKRARYFLGKRDADEINEEKRYRYFLGKRDDLMKRRYYFLGKRPAMLDDDFEKRRRFFLGKRRRFFLGKRDSTYNN
NDLGPQLGGFGESNNLEDTSQNGLRDFIQKRMRYFLGKRSDELDGQNLETDKRFARHFLGR*

LRF

MEVTTYKVAIAVLMFVCLMISDSQCGIPLNSFLSASNGKLERDADDVDNFDEETANIQGVRGQDLRKLILKKLRFRDLDDQSPPSLLESIQFPVKRDQDNLV
DKVAALLSGLKVRQISDSPSVRMPSLRFG*

LRFamide

MKCLLKSTARTTLGLLTLCIALEAFPYICSDCFPDDERCILKCSLQGQETNNLDSMSNVNSNRKRFQQQLRFGKRGPVGVTDQANDLEESFRPQLRFGKRSR
LPSKRRFMSHMRFGKRDFQFDYKSLQPQERSGRIPDAEISSLDTPRSSTGVLEIPAIFYGQGQEKRFRPQGRFGKRLRPGYETISETNNLPLYKGSPQTTSKEEL
TFDFDDMAVIGYQLPEK*

LRYamide

MHGQIMLPVILCLISMLSSFTDATDASGEVPISSGNLLREILESRDFTDVEKEILKYIIFSDVKDFVERKEASAESREDSDSGIERKRSHLWAYRQNNIPIQTRVA
FGRQLMRNSNGHGSNSNLLRYGK*

Luqin

MKLAEIITVLCVVFFVTLTVGDGAPQWRPQGRFGKRGDQRQYPLSWQPDSESDIDVYPVVERRSDTESDSEKTLKLLEKLCVESSLPGLFRCYRRKRSAAV
GEHDRS*

Lymnokinin/leucokinin

MGFNVAEMLPIVIFTTYVLISQRTLKAESRIVCSKVDTNNPSDKIIRFWIRRQRQQTLNPQAELKRPNFHPWAGKRSEQNSQEDFSDVFRRAFHAWGGKRSE
TNIPANFQDVIRRAFHAWGGKRLAGLQNLNSDFINIGPENIPLNSGKNEISDDKALPEKDNIQPYTSDGDVLSTNKLDIKRDFGAWGGR*

MIP 1

MKFLETCILALMLITQHILADGPGAAGSDAADRLEQYDGNSGTDILAQEIKRARIPQFVGKRDYFANDDLDLSDDSQNMPIEVAQAIERNLLSSLLGGSNEG
LPDGSEGAAQSEYFGRQTRYTPSMVGRRSKWVPKFVGKRRGPLFVGRRRSPYFVGKRTSQTDNDLAAEKRRNPMFVGKRADPVLVARGLSKPMFVGRRG
HPMFVGRRTASGDLDSSIFMGKR*

MIP 2

SSIFMGKRSVENLKSGQGLTSRRKRSVDKTTEHVAINTATIKAINQNKARQRFHSKVKRKKRDNYSKRDRQKRDSDFILDKDRRNFDAPFFVGKRGSNHFE
KLSATSSRITTLNSHDSKMPVLVGKRNREFNIPDFVGKRSQHPSDMLRFLERQTTSRITQLNNLNPPTLNQFPSSQYKRYSPHHFIGKRYPPPNFIGKRYSPPDF
IGKRYSPPNFISKRYSTPDFIGKRYSSPDFIGKRYSPHDFIGKRYSPND

Myomodulin

MKYILPIILLLHCHQLITGSTDNDNTSEENGSPLNRVRR**GGLSMLRL**GRGLQMLRLGKRSMPMNRIGRSLDTLSSDELKYLLVSVLGDKFNHRRQVPLPRY
GREDREDAELQWLLEHIGSDRVNTDESSEGLYDLDDESPRQIRLAPRPGRFRRSTDEQDKQDATQKGAYIQDVEEDKNEEEKAIPLPRVGRILYGGERALPLP
RLGRDEMYDYVYTLEPAKDSEDSDVDKRGMHMLRLGR**GMNMLRL**GKR**PMSMLRL**GRSEIQQEANKGDDKRSLSMLRLGKRLRMLRLGKRPDDGDRS
LRMMRLGKKDVDDSGSDSEHSVETRGMHMLRLGRNVYK*

NdWFamide

MTVIVLLFCSILMSSVLLSNANWYGKRGSEVDDAFQRYLEGRLVKSIGSRHSDDREILFKISKLLQEWQLKNKANEVAEVTSYDRK*

NKY

MTQGTSLLLVVFANFFVVQCYGSYLPGALGLSKSNEASNYIDSVITEEEKAFYAKVIQKLMDRAAALEEEVDGHNGDNFDSDGDLSSSSDLSGLKRSMDKR
KVFWQPLGYVPASMRMSPNNKHKASQKDVGRKGFRYGK*

NPF

MQKFVLTTVLIVCALLVSQVTCQEAMLEPPDRPHSFRTPDQLRSYLRALNEYYSIVGRPRFGRSVNKRSLESSLFNTEDLNTGDYPAFEDERDLYI*

Opioid

MIRTIGFVLVYVTVYVTAVKEVSIPVSDMSEGTRCDKDLDKAGTCFQCSRLPEEIAVQLTTCCMEDKAYDVCERCVGNPKACLMDAYRVTGSDYSQDASKD
TSEEMANIEYPDDPEIPLDKRFGTTFMGSSRYGYPRKRYGMLFLGRNKNRGGYRYGSRGKRYGTLNLGSGKGLMYRYGSDIKRADSSDEESMNKRYGTLN
LGSGRGLRYRYGRYGKRSDDDDQDDNNESFDDNNDDTEQIEKR**YGTLFMSRNG**GRKRYGSLFMGKSRNW*

Pedal peptide 1

NGGMSSFAKR**RFDSISGDGGMSTFA**KRRFDSISGNGKLSGFAKRPLDSISGNSGISGLMKR**RFDSIDGSAGLEGFN**KR**KFDSISGNGGLRGFS**KKNFDSID
GGAAFQGFAKK**SEQDSDTDEERNSL**SKR**RMDSIVGGGGFGRFH**KRNFDSIDGDGSIMGFAKKSFDSIDGGSLNAFVKKDDEA*

Pedal peptide 2

MLTGTAAMWLLTIALLLVCGRINGLQADHELDKRDADQDVSVDELPVDEKR**QFDSISGNSGLSGFA**KKTFDSISGGHGMPSFAKRTLDSISGNGGISSFAKR
KFDSISGNGGMSAFAKRNFDSISGNGGMSSFAKRRFDSISGN

PFVx7a

MLWPLCLFLSVTFATSNASIYYEDNREHILDQGVEEPGSSDGVVDDGPYSFKERPEKDEGLTYAPRYYAQFPVDDMEQPIVIGGKGYTFGRRPNVYGGYGLG
KQNGQQDEYILDDGEPLYNFNDRGDKMATNKRYFVFRGGRHQLGKRPFVFGSGGYFGKRDDDDDTDDNKATVNKR**PFVMRGGYNF**GKRDSGEPGETL
QKR**PFVLSGGYNF**GKRDNDEDRVQKRPFVLGGGYKIGKR**PFILGGGYKF**GKRNIDDGEQINTIKRLF**VLGGGYKF**GKGPFA**FGSGYKF**GKREVGDDDV
QKRPFILGSGYDFGKRFFAMREGHIFAKRDMKNDEGNEIEKRPFVLGGRYKFGKR**PFVFGSGYKF**GKRGMGEFEDDEEIEKRPFVLGSGYNIGKR**PFVMR**
GGYNFGKRGLDNEGDIVEKR**PFVFGSGYKF**GKR**PFVLGSGYQF**GKRADPETEIEKRPFVLSGG

PKYMDT

MDINVTAVLSMFLSLLLIMPALSIPLDSQETQEHLEEKRPKYMDTRELGDMMQELVYNALKELVSKGKVNEEVFPTPEGFVEKSEAPSETNSVDKRRRHLSY
CLRRSGPNFVPYPCYKYGGRR*

Pleurin

MSPHSILTLLTTFAIIQLAKA**IFYTNKEGNDFPRI**GKRRHLYQSEAWSRDSVPSDGLPGETDRQMEPMYNQDEGEIPITSRREHQNPMSSYSKAMYYRALGL
NGYRAFSKNKVQASPNNGIRGTRTYY*

QSamide

MICTKVVAKVLLVCAVFGCIACGRSVEGKLKRLRR**QTGDVKTAEYFARL**ALERRPTGCDLIGCGLIDIVASGKKRDGRTASAYQGQIDDESRAKLIAMLLTG
SAETRRK*

RSamide

MYVQLFVCLCCCVVMVTPRATTPLEHRVRRQAADFKIAQYRACMSVNSFYNKDCRNIGWGLVDVLRSGRRKKSDLAPLQLIMDELTNDNNDELDDTYL*

SCAP

MVSGLLSGCVLLVAFSLYTEGKAVSLDRQKR**APNFLAYPRG**GRSSNPMAFSRDAR**AMFSYPRL**GRSGAGFPADVLDGLGCCNMGVKSINGMAMCSASEC
CEGLQEYRDVKKPFMFTLCVWDNSAEEDKA*

SYGAGamide

MQPNIYVCSYVAVVVVILSTIAHCYPSKQIDLFTDSLPDDSYFFEDRSDRRASLAAPVLSLSSRTYHPLQQLIKRGYQNKRYSLNDLIARLRAMVGSDEIRHS
GRSSYLRFGAGGKR*

Tachykinin

MVNLDSITIVGLLVVAVFGVEAFTENSLWSQSDESPDTYGHMYDSEESVPQYRDNTRVTQLVESPEEAFRMIEAMKARTRRRMSPFSAMRGKK**SVSESQEK**
HNMDKAIRGKRTIQDESDNGSYDLQKR**AAYFKAMKSSLR**GKRMVPAYSSPVSEFEYQLLLQKVLAAAMSLSGNRNGDEQSMMGKR**NYGFHALR**GR*

VAKKSPH

MVPLLLIAALGLVNGASYSDILSELNGLYDLDPGYYEDNLVSRSSPGDWGMSYRDAPLNEPELLGEAALRDQEYLEQSPLWGYQSMSGGTGGGKENPKEV
KTDKVLPAYCNPPNPCPIGYKADDNCLETFANTPDNNRNLMKQQDCPCDSEHMLSCPNDKGRINTQSQSTDDISNMLQSIDLADSMEDNNPYFGSTEDRIS
RVAKKSPHMIKKRTPEEFTGEVRSGEPKRLAKKSGTEYKNQQIFQDLLEGEY*

Vamide

MNVRHRNGITNVLMSMCFLIFFYDVMALPGKQVSDGGRKHITSGDFDSLNTEEIPSELFLDINDDDGIDDLEHQYEVQQFDDPRYVTVEEPGVGKRFLGRW
RNLNWKLRQRELNKDDVPVGKRFFGRWMNRNYILSKLEAERKNQVGKRFLGRWRNRNYLLEKMKDE*

Wx3Ya

MDMSTLRTAYTMCNVLLVILGVTLLSQGTIGLPLHTEEQGPLLSLLTGKQHPDIYDRLLENYMKTLETRDEPYSGEYVVPDKRLRQGWNIAYGKRR**SNKWS**
IAYGKRDHASPLESEGIFSDEVIPLVRSYPEMSKRQQGWHIAYG*
