# Supplementary figures and images for: Identification and Characterization of Neuropeptides by Transcriptome and Proteome Analyses in a Bivalve Mollusc Patinopecten yessoensis
Source: Front Genet. 2018 Jun 5;9:197. doi: 10.3389/fgene.2018.00197 (PMC5996578; doi:10.3389/fgene.2018.00197)

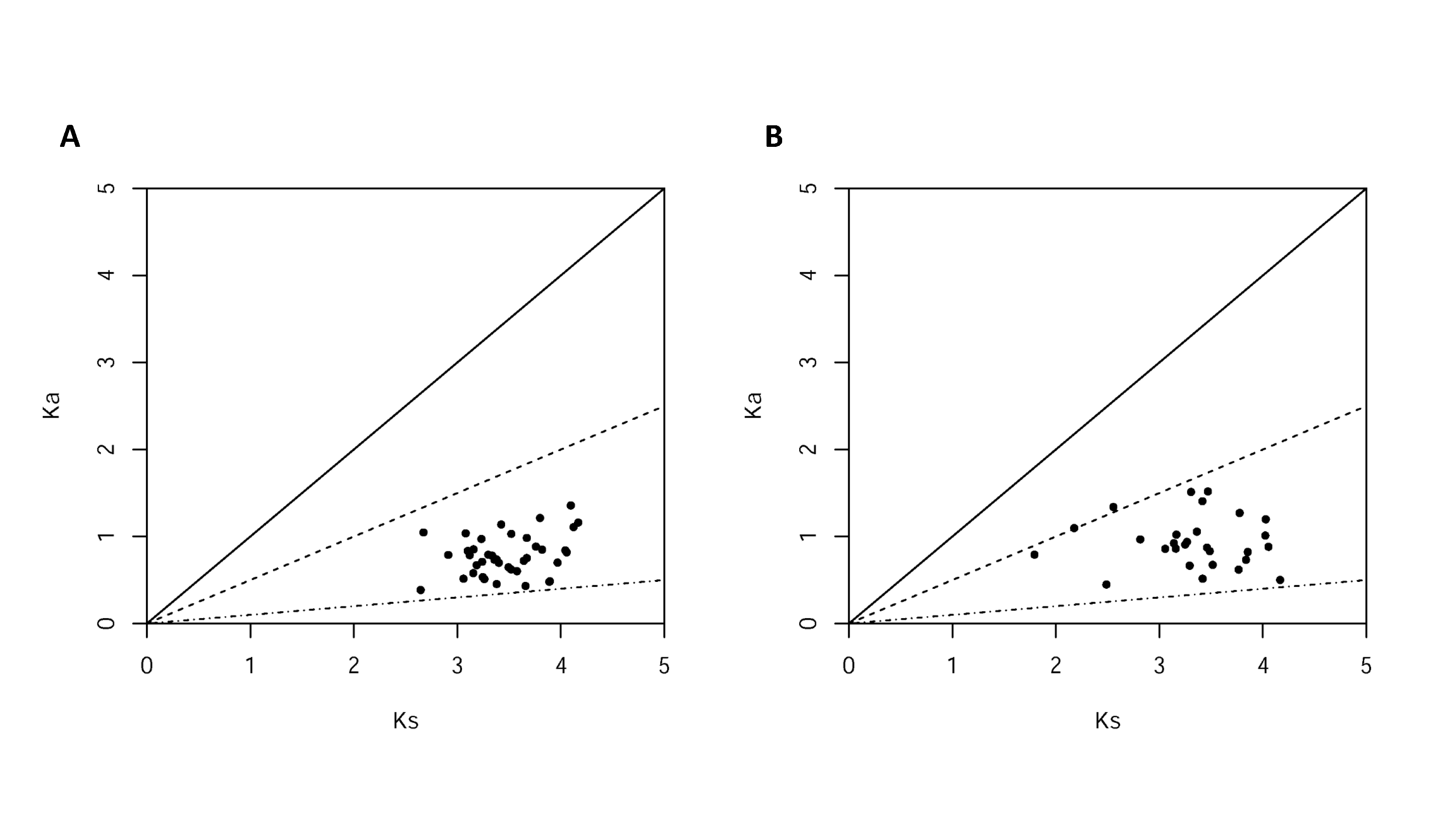

Supplement: FIGURE S2 — Distribution of Ka/Ks ratios of neuropeptide genes between P. yessoensis and two related molluscs, C. gigas (A) and D. reticulatum (B). The solid line shows the threshold of Ka/Ks = 1, the dashed line marked the threshold of Ka/Ks = 0.5, and the short dashed line represented threshold of Ka/Ks = 0.1. [file Image_2.TIFF]
